# Emergency Medical Service Directors’ Protocols for Exertional Heat Stroke

**DOI:** 10.3390/medicina56100494

**Published:** 2020-09-24

**Authors:** Michael R. Szymanski, Samantha E. Scarneo-Miller, M. Seth Smith, Michelle L. Bruner, Douglas J. Casa

**Affiliations:** 1 Korey Stringer Institute, Department of Kinesiology, University of Connecticut, Storrs, CT 06269, USA; samantha.scarneomiller@hsc.wvu.edu (S.E.S.-M.); douglas.casa@uconn.edu (D.J.C.); 2 Division of Athletic Training, School of Medicine, West Virginia University, Morgantown, WV 26506, USA; 3 Department of Orthopedics and Rehabilitation, University of Florida, Gainesville, FL 32607, USA; smithms@ortho.ufl.edu (M.S.S.); bruneml@ortho.ufl.edu (M.L.B.)

**Keywords:** cool-first transport-second, EHS, heat illness, cold-water immersion

## Abstract

*Background and Objectives:* Emergency Medical Service (EMS) protocols vary widely and may not implement best practices for exertional heat stroke (EHS). EHS is 100% survivable if best practices are implemented within 30 min. The purpose of this study is to compare EMS protocols to best practices for recognizing and treating EHS. *Materials and Methods:* Individuals (*n* = 1350) serving as EMS Medical or Physician Director were invited to complete a survey. The questions related to the EHS protocols for their EMS service. 145 individuals completed the survey (response rate = 10.74%). Chi-Squared Tests of Associations (χ^2^) with 95% confidence intervals (CI) were calculated. Prevalence ratios (PR) with 95% CI were calculated to determine the prevalence of implementing best practices based on location, working with an athletic trainer, number of EHS cases, and years of directing. All PRs whose 95% CIs excluded 1.00 were considered statistically significant; Chi-Squared values with *p* values < 0.05 were considered statistically significant. *Results:* A majority of the respondents reported not using rectal thermometry for the diagnosis of EHS (*n* = 102, 77.93%) and not using cold water immersion for the treatment of EHS (*n* = 102, 70.34%). If working with an athletic trainer, EMS is more likely to implement best-practice treatment (i.e., cold-water immersion and cool-first transport-second) (69.6% vs. 36.9%, χ^2^ = 8.480, *p* < 0.004, PR = 3.15, 95% CI = 1.38, 7.18). *Conclusions:* These findings demonstrate a lack of implementation of best-practice standards for EHS by EMS. Working with an athletic trainer appears to increase the likelihood of following best practices. Efforts should be made to improve EMS providers’ implementation of best-practice standards for the diagnosis and management of EHS to optimize patient outcomes.

## 1. Introduction

Approximately 9000 exertional heat illness cases are treated each year in high school athletics [1]. Over 3332 deaths attributed to heat stroke were reported in the United States from 2006–2010 [2]. From 1995 to 2017, exertional heat stroke was responsible for 63 deaths in American football players. [3]. In the occupational setting there were 359 deaths reported in workers from 2000 to 2010 [4]. More recently, 79 heat-related deaths were reported in the occupational setting during the span of 2014 through 2016 [5] Exertional heat stroke (EHS) is characterized by an internal body temperature ≥40.5 °C (105 °F) accompanied by neurological dysfunction [6,7]. The best practice for recognizing and assessing for EHS includes examining the mental status of the patient for central nervous system dysfunction and obtaining a rectal temperature [6,7,8,9]. Rapid cooling in less than 30 min from the time of collapse is necessary to prevent critical cell damage [9,10]. The gold-standard or best practice for the treatment of EHS is cold-water immersion (CWI) [2,7,8,9,11]. Given that EHS is 100% survivable within 30 min of collapse, it is imperative that cooling begins as soon as possible and should not cease until the internal body temperature has decreased to safe levels (e.g., 102 °F). As such, cooling should be done prior to transport to the hospital to improve the outcome of the patient [9,10,12,13,14]. In a retrospective study of 274 EHS cases during the Falmouth Road Race, the authors reported a 100% survival rate and 93% of the patients were cleared for home discharge by a medical tent physician when immediate onsite CWI immersion was implemented [12].

The signs and symptoms of EHS can mimic a broad array of other diagnoses and therefore be difficult to differentiate from other medical conditions. The differential diagnosis for an athlete who collapses from physical exertion in warm environments can include, but is not limited to, cardiac arrhythmia, hyponatremia, hypoglycemia, traumatic head injury, and hyperthermia. One of the most common misconceptions with EHS patients is that they will present hot dry skin. However, most EHS patients will present hot wet skin [9], altered central nervous system (CNS) function, and behavioral changes. The most accurate and feasible way to obtain a valid internal body temperature is using rectal thermometry. While gastrointestinal capsules may also be valid, they must be ingested at least one hour beforehand, which makes this method of body core temperature evaluation difficult for certain sporting activities (i.e., mass participation events) [15]. Alternative methods of measuring internal body temperature (temporal, tympanic, sublingual, and axillary) do not accurately measure core body temperature in a person who has been exercising [15,16,17,18]. It is also important to note that these other methods of measuring internal body temperature are not correlated to rectal temperature and should not be used in place of, or as a substitute for, a rectal temperature. Once a diagnosis of EHS is established, rapid cooling should begin immediately. The gold standard for treatment of a patient suffering from EHS is cold-water immersion (CWI) initiated within 30 min of collapse for best long-term outcomes [2,7,8,9,11]. Treatments such as cold packs applied to the axilla, neck, and groin are ineffective and should not be used alone for the rapid cooling of patients [2,11]. Nonetheless, these ineffective cooling techniques continue to be the recommended as the sole cooling modality in multiple state emergency medical service (EMS) protocols, despite being out dated methods. Further, because cell damage occurs within 30 min of an internal body temperature over 105°F, cooling should be initiated immediately on site until the internal body temperature reaches 102°F, and then transported once the patient is no longer hyperthermic [9,10,12,13,14].

A recent consensus statement outlined the care necessary for the prehospital recognition (i.e., rectal temperature) and treatment for EHS (i.e., CWI) [9]. This document is the first to outline the aforementioned best practices, along with techniques to integrate them within EMS systems. A majority of EMS protocols require the prompt evaluation of a patient followed by immediate transport to the local medical facility. However, the information outlined in the consensus statement states the need for cooling to be done on-site prior to transportation to the hospital [9]. The document also provides information for large athletic event planning and communication with EMS staff and other healthcare professionals, which can improve the overall outcome of EHS patients. Best practices for EHS (i.e., rectal temperature, CWI, cool-first transport-second) should be implemented into state and local EMS protocols to ensure the survival rate and best outcome for these patients. Despite this consensus statement, we believe that only Connecticut EMS protocols include rectal thermometry, CWI, and cool-first transport-second as the required protocol for EMS providers.

To gain a better understanding of current EMS protocols, we surveyed EMS directors across the country. The purpose of this study was to compare EMS protocols against variables such as the best practice standards for EHS (i.e., rectal temperature, CWI, cool first transport second), location of the respondent, number of EHS cases treated, and working with an athletic trainer (AT). We hypothesize that most EMS protocols do not follow best practices and that working with an AT will increase the likelihood of following best practices for EHS.

## 2. Methods

### 2.1. Study Design

This was a cross-sectional survey study design. The study and all procedures were reviewed and deemed as exempt by the University of Florida’s Institutional Review Board. Due to the anonymous nature of data collection, an information sheet was displayed to the potential participants, and they were asked to click “next” if they agreed to participate.

### 2.2. Participants

Individuals currently serving as an EMS Medical or Physician Director in the United States were eligible to complete the anonymous online survey.

### 2.3. Procedure

A letter containing a link to the web-based survey was mailed to 1350 members of the National Association of EMS Physicians (NAEMSP) over a 13-week period. Through the agreement with NAEMSP, each member received only one letter notifying them of the study. Survey responses were collected over a 6-month period and stored using the Research Electronic Data Capture (REDCap) [19] application at the University of Florida. The survey contained 2 pre-screening questions to confirm respondents read the waiver of consent and verify if they currently served as an EMS Medical Director. Only those who read the waiver of consent and were current EMS Medical Directors were able to proceed with the survey. During the 6-month survey period, 174 respondents completed the pre-screening questions to ensure they read the consent document and to confirm they currently serve as an EMS Medical Director (valid response rate = 12.88%). Ineligible responses (e.g., not current EMS Medical Director) (*n* = 18) and incomplete responses (defined as those with less than 100% of questions completed), (*n* = 11) were removed. A total of 145 responses were included in the analysis (true response rate = 10.74%).

### 2.4. Survey Instrument

Previously published surveys investigating AT management of EHS were used as a guide to create the EMS survey [20,21]. An anonymous, 9-question survey investigating EMS Medical Directors’ practices regarding the management of EHS was administered through the secure, web-based REDCap application. Respondents indicated which general region they resided in (Northeast, South, Midwest, West) and how many years of experience they had as EMS Medical Directors. The survey contained three questions regarding local EMS awareness of classic heat stroke vs. EHS, if continuing medical education credit was offered for EHS courses, and if they work with ATs for the medical care of athletes. Respondents were also asked to estimate the number of EHS cases that occur in their region annually, along with treatment protocols for EHS (Appendix A).

### 2.5. Statistical Analysis

We summarized the responses of the questions through frequency distribution. A 95% confidence interval (CI) for the prevalence ratios (PR) and Chi-Squared Tests of Associations (χ^2^) were calculated to determine the likelihood of implementing best practices based on location, working with an AT, number of EHS cases, and years of directing. Additional variables were created to compile a comprehensive understanding of the current adoption of best practices. Best practices for the diagnosis and treatment of EHS were defined as performing rectal temperature, the use of cold-water immersion for the treatment of EHS. and cool-first transport-second. The three questions in the survey/questionnaire that asked this were combined into a singular variable termed Best-Practice for EHS (BPehs).

All PRs whose 95% CIs excluded 1.00 were considered statistically significant; χ^2^ values with *p* values < 0.05 were considered statistically significant. All statistical analyses were computed using SPSS (SPSS Statistics version 25, IBM Corp., Armonk, NY, USA).

## 3. Results

**Figure 1:**
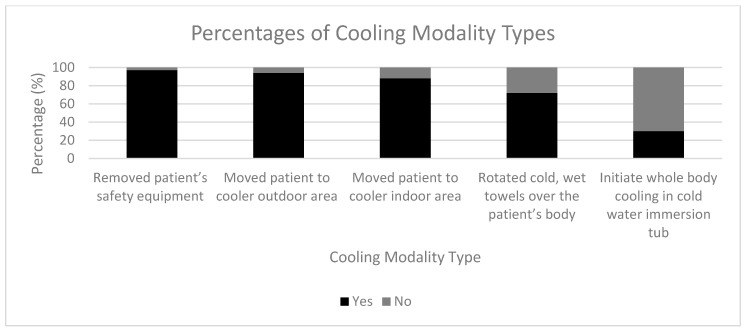
Types of cooling modalities used for the treatment of exertional heat stroke (EHS). Participants were asked to answer yes or no to the different types of cooling modalities their Emergency Medical Service (EMS) protocols outlined for the treatment of EHS. Data shown as percentages (%) of the participants’ answers for each cooling modality.

**Figure 2:**
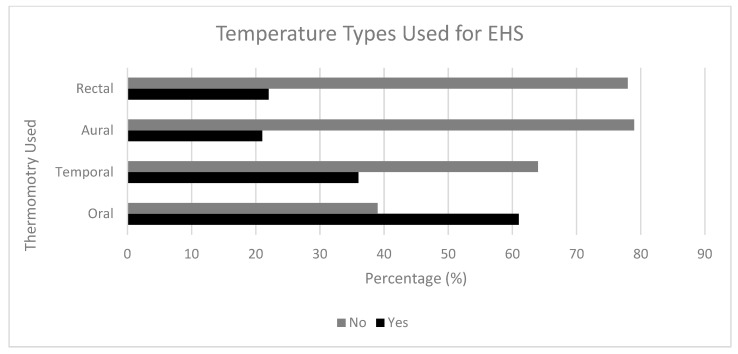
Types of thermometry used for the diagnosis of EHS. Participants were asked to answer yes or no to the different types of thermometry used in their EMS protocols outlined for the diagnosis/recognition of EHS. Data shown as percentages (%) of the participants’ answers for each thermometry used.

The frequencies of each location as well as the number of years directing are summarized in Table 1. A majority of respondents stated they had a specific treatment protocol for EHS (*n* = 86, 59.31%). Additional responses to the questions are found in Table 2. The northeast location was more likely to use BPehs compared to other locations (20.0% vs. 7.5%, PR = 2.34 (95% CI = 1.04, 5.26)). The other locations (i.e., South, Mid-West, West) were not statistically significant for the use of best practices. If the use of rectal temperature was reported, then they were more likely to implement CWI and cool-first transport-second, compared to not using rectal thermometry (43.8% vs. 8.0%, χ^2^ = 23.93, *p* < 0.001, PR = 4.13 (2.41, 7.06)). The percentages for different cooling modalities used for the treatment of EHS are summarized in Figure 1, with 30% of participants indicating that they use CWI for the treatment of EHS. The percentages for temperature types used to recognize EHS are summarized in Figure 2, with 22% of participants indicating that they use rectal thermometry for recognizing EHS. 

Respondents from the South reported a higher prevalence of working with an AT compared to the other locations (52.9% vs. 36.2%, PR = 1.55 (1.00, 2.41)). If working with an AT, EMS reported being more likely to implement best-practice treatment (i.e., CWI and cool-first transport-second), compared with not working with an AT (69.6% vs. 36.9%, χ^2^ = 8.48, *p* = 0.004, PR = 3.15 (1.38, 7.18)). Respondents from the South were also more likely to have an EHS protocol when compared to the other 3 locations (72.5% vs. 52.1%, χ^2^ = 5.71, *p* = 0.02, PR = 1.81 (1.08, 3.03). The northeast was least likely to have an EHS protocol when compared with the rest of the country (40.0% vs. 63.3%, χ^2^ = 4.67, *p* < 0.03, PR = 0.46 (0.22, 0.95)).

## 4. Discussion

Given death from EHS is 100% preventable with prompt recognition and immediate treatment, it is imperative to understand the current management practices of EHS by EMS across the country. Important findings of our study included a lack of implementation by most EMS providers of the gold-standard method of thermometry for EHS (i.e., rectal temperature) (Figure 1) and showed that most EMS providers do not use the gold-standard for treatment of EHS (i.e., CWI and cool-first transport-second) (Figure 2). In addition, when working with ATs, EMS providers may be more likely to use best practices for recognizing and treating EHS. Lack of preparedness for EHS management may lead to an increase in patient complications and mortality.

Our findings indicate when EMS report using rectal temperature in a suspected EHS case, they are more likely (PR = 4.13) to use CWI and cool-first transport-second. This may be due to the EMS providers being more familiar with the best practice standards for EHS (i.e., rectal temperature, CWI, cool-first transport-second) or to the fact that they have rectal temperature incorporated into their EHS protocol. Our study highlights a worrisome finding in that a majority of the participants do not follow the recommendations described in the consensus statement on the prehospital care of EHS. The consensus statement highlights the importance of using rectal temperature for assessment of potential EHS. In addition, it describes the use of CWI for appropriate treatment, as well as cool-first and transport-second [9]. EMS directors dictate which treatment and management strategies are used, which may be based on their current region/state protocol or their current knowledge on the appropriate management of EHS. If best practices for EHS are not specified in a state or region protocol, then it is unlikely that these practices will be used in the evaluation and treatment of EHS. This idea emphasizes the need for policy/protocol changes that adhere to the best practices for the management of EHS that are described in the current literature, to ensure the optimal outcomes in patients who suffer from EHS.

Our study highlights the current information regarding the care of EHS provided for continuing medical education (CME) is outdated and inaccurate. Therefore, the need for updated educational materials for EMS training and retraining is paramount. For example, the book *Prehospital Emergency Care* (11th Edition) emphasizes not using cold water to cool the patient, promoting instead the utilization of ice packs placed on major arteries, along with transport without delay [22]. The book [22] also mentions that hot dry skin is a sign of EHS, which is incorrect, as patients typically present hot and wet skin [7]. Unfortunately, this outdated information does not adequately prepare EMS providers to properly manage EHS. This type of out-of-date education contradicts the current best-practice standards for treating EHS (i.e., CWI and cool-first transport-second) and may lead to further complications, including increased patient mortality [9,12]. Aggressive cooling while transporting to the nearest hospital may prove challenging, given that a typical ambulance does not have the capabilities to host a cold-water immersion tub. Continued research is necessary to identify other effective cooling mechanisms that can be used en-route to the emergency department for the adequate cooling of patients who suffer from EHS. If the local EMS protocol for EHS does not allow for a delay in transport in order to treat, then a cooling modality as effective as CWI should be established, so the patient does not remain hyperthermic for a prolonged period of time, ultimately leading to increased morbidity and mortality.

We also identified regional differences regarding EHS protocols, indicating that the respondents from the south were more likely to have an EHS protocol compared to the rest of the country. This may be due in part to the fact that the south has an overall hotter climate and thus may facilitate a higher perceived susceptibility for EHS cases. For example, the 90th percentile warm season (May–September) maximum daily wet-bulb globe temperatures (WBGT) for the northernmost states is ~28–30 °C, whereas for the southernmost states the WBGT is ~34–36 °C [23]. Our findings also suggest respondents in the south are almost two times more likely to work with ATs when compared to the rest of the country. The increased collaboration with ATs, another healthcare professional, may provide another reason why EMS providers in the south are more likely to have EHS protocols implemented. We also found that the Northeast is least likely to have an EHS protocol when compared to the other regions in the country. Again, this may be related to the climate of the Northeast, and the EMS directors who write the policies may not feel that it is as important due to the perceived temperate climate. However, it is important to recognize that EHS can occur when the environmental conditions are above the normal for that region. In other words, a hot day in Georgia and a hot day in Maine may be vastly different. However, the abnormally hot day in Maine may contribute to an EHS injury just as much as the hot day in Georgia [23]. In contrast, the Northeast was over twice as likely to use best practices for EHS (i.e., rectal temp, CWI, cool-first transport-second) when compared to the rest of the country, even though they were least likely to have an EHS protocol implemented.

The positive finding of increased likelihood of the use of best practices when working with ATs highlights the importance of increased collaboration between EMS providers and ATs. ATs are trained to prevent, recognize, and treat various injuries and aliments, especially in emergency care settings. The collaboration between ATs and EMS providers will ensure that best practices are used to improve the best outcomes for the patient. This is especially important as healthcare professionals become more commonplace outside of the traditional healthcare setting (i.e., secondary school setting, occupational settings, and military settings). While these findings were encouraging, it is important to note that most recent literature on ATs also depicts that a relatively low number of ATs have access to a rectal thermometer [24]. Further, only 18.6% of ATs used rectal thermometry to measure core body temperature in suspected EHS cases [21]. The lack of rectal thermometry usage among athletic trainers may be due to a number of barriers including a lack of training, equipment, and perceived invasiveness [21].

### Limitations

One area that was not addressed in our study was examining how age, sex, and years out from training of EMS directors affect the management of EHS. It may be plausible that younger EMS Medical Directors are more familiar with the current literature and more likely to implement the new evidence in their protocols. Moreover, by examining the differences (e.g., self interest in learning best practices, mandatory CMEs, educational differences, resources, etc.) between the EMS directors who currently employ best practices for EHS and those who do not can provide insights as to what barriers must be overcome in order for all EMS directors to implement best practices for EHS into their protocols. There are other limitations worth noting for our study. First, there was a low response rate. There were 145 respondents with a true response rate (10.74%). Second, we had limited access to the target population through the distribution method with the NAEMSP.

## 5. Conclusions

In conclusion, our study underlines that most EMS Medical Directors do not incorporate best practices for the recognition and treatment of EHS. We show the importance of collaboration with ATs and how that can increase the likelihood of using best practices for EHS. Given that the current educational literature is outdated in the EMS world, improved educational and CME trainings regarding the signs and symptoms of EHS (e.g., hot dry skin vs. hot wet skin) and the best practices for the management of EHS are clearly needed. Future research is warranted to evaluate how best practices can be incorporated and what factors are present that could influence the implementation of these practices in order to improve patient outcomes.

## Figures and Tables

**Table 1:** Respondent Demographics.

Location		
	Northeast	25 (17.2%)
	South	51 (35.2%)
	Midwest	43 (29.7%)
	West	26 (17.9%)
**Number of Years Directing**		
	<5	43 (29.7%)
	5–9	32 (22.1%)
	10–14	16 (11.0%)
	15–19	26 (17.9%)
	20–24	11 (7.6%)
	25+	17 (11.7%)
**Approximate Number of EHS Cases**		
	<5	52 (35.9%)
	5–15	51 (35.2%)
	15–25	22 (15.2%)
	25–35	3 (2.0%)
	>35	17 (11.7%)
**EHS Management Strategy/Protocol**		
	Cool on-site	31 (21.4%)
	Cool en-route	91 (62.8%)
	None	23 (15.8%)
**Work with an Athletic Trainer**		
	Yes	61 (42.9%)
	No	71 (49.0%)
	Unsure	13 (8.1%)

EHS, exertional heat stroke.

**Table 2:** EHS Protocol Responses by Region.

Are Your EMS Service Members Aware of the Different Clinical Presentations of Classic vs. Exertional Heat Stroke?		
	Yes	No
**NE**	20 (18.02%)	5 (14.70%)
**S**	45 (40.54%)	6 (17.65%)
**MW**	34 (30.63%)	9 (26.47%)
**W**	12 (10.81%)	14 (41.18%)
**TOTAL**	111 (76.55%)	34 (23.45%)
**Does your EMS program offer continuing medical education for exertional heat stroke?**		
	Yes	No
**NE**	15 (16.67%)	10 (18.18%)
**S**	35 (38.89%)	16 (29.09%)
**MW**	26 (28.89%)	17 (30.91%)
**W**	14 (15.56%)	12 (21.82%)
**TOTAL**	90 (62.07%)	55 (37.93%)
**Do you have a specific treatment protocol for exertional heat stroke?**		
	Yes	No
**NE**	10 (11.63%)	15 (25.42%)
**S**	37 (43.02%)	14 (23.73%)
**MW**	25 (29.07%)	18 (30.51%)
**W**	14 (16.28%)	12 (20.34%)
**TOTAL**	86 (59.31%)	59 (40.69%)
**Do you initiate whole body cooling in cold water immersion tub?**		
	Yes	No
**NE**	8 (18.61%)	17 (16.67%)
**S**	15 (34.88%)	36 (35.29%)
**MW**	13 (30.23%)	30 (29.41%)
**W**	7 (16.28%)	19 (18.63%)
**TOTAL**	43 (29.66%)	102 (70.34%)

NE, Northeast; S, South; MW, Midwest; W, West.

## Appendix A. Survey Questions

In which region of the country are you located?

(a) Northeast
(b) South
(c) Midwest
(d) West

How many years have you been an EMS director?

(a) <5
(b) 5–9
(c) 10–14
(d) 15–19
(e) 20–24
(f) 25+

Are your EMS service members aware of the different clinical presentations of classic vs. exertional heat stroke?

(a) No
(b) Yes

Does your EMS program offer continuing medical education (CME) for exertional heat stroke?

(a) No
(b) Yes

Does your EMS service work with athletic trainers in the medical care of athletes?

(a) No
(b) Yes
(c) Unsure

Do you have a specific treatment protocol for exertional heat stroke?

(a) No
(b) Yes

In regards to the management of exertional heat stroke, which of the following strategies are used by your EMS service members:

Removed patient’s safety equipment

(a) No
(b) Yes

Moved patient to cooler outdoor area (into shade, etc)

(a) No
(b) Yes

Moved patient to cooler indoor area

(a) No
(b) Yes

Rotated cold, wet towels over the patient’s body

(a) No
(b) Yes

Initiate whole body cooling in cold water immersion tub

(a) No
(b) Yes

Temperature taken:

Oral

(a) No
(b) Yes

Temporal

(a) No
(b) Yes

Aural

(a) No
(b) Yes

Rectal

(a) No
(b) Yes

Placed ice bags on the body during transport to emergency department:

Neck

(a) No
(b) Yes

Axilla

(a) No
(b) Yes

Groin

(a) No
(b) Yes

If you had to summarize your EMS service’s overall exertional heat stroke management strategy/protocol in one statement, which one of the following would you select?

(answer only one of the following)

(a) Cool en-route
(b) Cool on-site until temp hits 102
(c) No current EHS protocol

Approximately how many cases of exertional heat stroke occur in your region annually?

(a) <5
(b) 5–15
(c) 15–25
(d) 25–35
(e) >35

## References

[1] Kerr Z.Y., Casa D.J., Marshall S.W., Comstock R.D. (2013). Epidemiology of exertional heat illness among, U.S. high school athletes. Am. J. Prev. Med..

[2] Gaudio F.G., Grissom C.K. (2016). Cooling Methods in Heat Stroke. J. Emerg. Med..

[3] Adams W.M. (2019). Exertional Heat Stroke within Secondary School Athletics. Curr. Sports Med. Rep..

[4] Gubernot D.M., Anderson G.B., Hunting K.L. (2015). Characterizing occupational heat-related mortality in the United States, 2000–2010: An analysis using the Census of Fatal Occupational Injuries database. Am. J. Ind. Med..

[5] Roelofs C. (2018). Without Warning: Worker Deaths From Heat 2014–2016. New Solut..

[6] Armstrong L.E., Casa D.J., Millard-Stafford M., Moran D.S., Pyne S.W., Roberts W.O., American College of Sports Medicine (2007). American College of Sports Medicine position stand. Exertional heat illness during training and competition. Med. Sci. Sports Exerc..

[7] Casa D.J., DeMartini J.K., Bergeron M.F., Csillan D., Eichner E.R., Lopez R.M., Ferrara M.S., Miller K.C., O’Connor F., Sawka M.N. (2015). National Athletic Trainers’ Association Position Statement: Exertional Heat Illnesses. J. Athl. Train..

[8] Casa D.J., Armstrong L.E., Ganio M.S., Yeargin S.W. (2005). Exertional heat stroke in competitive athletes. Curr. Sports Med. Rep..

[9] Belval L.N., Casa D.J., Adams W.M., Chiampas G.T., Holschen J.C., Hosokawa Y., Jardine J., Kane S.F., Labotz M., Lemieux R.S. (2018). Consensus Statement-Prehospital Care of Exertional Heat Stroke. Prehosp. Emerg. Care.

[10] Heled Y., Rav-Acha M., Shani Y., Epstein Y., Moran D.S. (2004). The “golden hour” for heatstroke treatment. Mil. Med..

[11] Casa D.J., McDermott B.P., Lee E.C., Yeargin S.W., Armstrong L.E., Maresh C.M. (2007). Cold water immersion: The gold standard for exertional heatstroke treatment. Exerc. Sport Sci. Rev..

[12] Demartini J.K., Casa D.J., Stearns R., Belval L., Crago A., Davis R., Jardine J.J. (2015). Effectiveness of cold water immersion in the treatment of exertional heat stroke at the Falmouth Road Race. Med. Sci. Sports Exerc..

[13] Sloan B.K., Kraft E.M., Clark D., Schmeissing S.W., Byrne B.C., Rusyniak D.E. (2015). On-site treatment of exertional heat stroke. Am. J. Sports Med..

[14] Zeller L., Novack V., Barski L., Jotkowitz A., Almog Y. (2011). Exertional heatstroke: Clinical characteristics, diagnostic and therapeutic considerations. Eur. J. Intern. Med..

[15] Casa D.J., Becker S.M., Ganio M.S., Brown C.M., Yeargin S.W., Roti M.W., Siegler J., Blowers J.A., Glaviano N.R., Huggins R.A. (2007). Validity of devices that assess body temperature during outdoor exercise in the heat. J. Athl. Train..

[16] Ronneberg K., Roberts W.O., McBean A.D., Center B.A. (2008). Temporal artery temperature measurements do not detect hyperthermic marathon runners. Med. Sci. Sports Exerc..

[17] Ganio M.S., Brown C.M., Casa D.J., Becker S.M., Yeargin S.W., McDermott B.P., Boots L.M., Boots L.M., Armstrong L.E., Maresh C.M. (2009). Validity and reliability of devices that assess body temperature during indoor exercise in the heat. J. Athl. Train..

[18] Moran D.S., Mendal L. (2002). Core temperature measurement: Methods and current insights. Sports Med..

[19] Harris P.A., Taylor R., Thielke R., Payne J., Gonzalez N., Conde J.G. (2009). Research electronic data capture (REDCap)—A metadata-driven methodology and workflow process for providing translational research informatics support. J. Biomed. Inform..

[20] Kerr Z.Y., Marshall S.W., Comstock R.D., Casa D.J. (2014). Exertional heat stroke management strategies in United States high school football. Am. J. Sports Med..

[21] Mazerolle S.M., Scruggs I.C., Casa D.J., Burton L.J., McDermott B.P., Armstrong L.E., Maresh C.M. (2010). Current knowledge, attitudes, and practices of certified athletic trainers regarding recognition and treatment of exertional heat stroke. J. Athl. Train..

[22] Mistovich J.J., Karren K.J., Werman H.A., Hafen B.Q. (2018). Prehospital Emergency Care.

[23] Grundstein A., Williams C., Phan M., Cooper E. (2015). Regional heat safety thresholds for athletics in the contiguous United States. Appl. Geogr..

[24] Scarneo S.E., DiStefano L.J., Stearns R.L., Register-Mihalik J.K., Denegar C.R., Casa D.J. (2019). Emergency Action Planning in Secondary School Athletics: A Comprehensive Evaluation of Current Adoption of Best Practice Standards. J. Athl. Train..

